# Real-time machine learning model to predict in-hospital cardiac arrest using heart rate variability in ICU

**DOI:** 10.1038/s41746-023-00960-2

**Published:** 2023-11-23

**Authors:** Hyeonhoon Lee, Hyun-Lim Yang, Ho Geol Ryu, Chul-Woo Jung, Youn Joung Cho, Soo Bin Yoon, Hyun-Kyu Yoon, Hyung-Chul Lee

**Affiliations:** 1 Department of Anesthesiology and Pain Medicine, Seoul National University Hospital, Seoul, Republic of Korea; 2 Department of Data Science Research, Innovative Medical Technology Research Institute, Seoul National University Hospital, Seoul, Republic of Korea; 3 Department of Medical Device Development Support, Innovative Medical Technology Research Institute, Seoul National University Hospital, Seoul, Republic of Korea; 4 Department of Anesthesiology and Pain Medicine, Seoul National University College of Medicine, Seoul National University Hospital, Seoul, Republic of Korea; 5 Department of Critical Care Medicine, Seoul National University Hospital, Seoul, Republic of Korea

**Keywords:** Risk factors, Outcomes research

## Abstract

Predicting in-hospital cardiac arrest in patients admitted to an intensive care unit (ICU) allows prompt interventions to improve patient outcomes. We developed and validated a machine learning-based real-time model for in-hospital cardiac arrest predictions using electrocardiogram (ECG)-based heart rate variability (HRV) measures. The HRV measures, including time/frequency domains and nonlinear measures, were calculated from 5 min epochs of ECG signals from ICU patients. A light gradient boosting machine (LGBM) algorithm was used to develop the proposed model for predicting in-hospital cardiac arrest within 0.5–24 h. The LGBM model using 33 HRV measures achieved an area under the receiver operating characteristic curve of 0.881 (95% CI: 0.875–0.887) and an area under the precision-recall curve of 0.104 (95% CI: 0.093–0.116). The most important feature was the baseline width of the triangular interpolation of the RR interval histogram. As our model uses only ECG data, it can be easily applied in clinical practice.

## Introduction

In-hospital cardiac arrest is a sudden and unexpected complication in patients admitted to intensive care units (ICUs). Despite advancements in critical care medicine, the incidence of cardiac arrest in ICU patients remains high, with reported rates ranging 0.5–7.8% upon hospital admission^1^. Early identification and rapid treatment are key to improving patient outcomes, but limited ICU resources and diverse causes of in-hospital cardiac arrest pose difficulties in preventing this life-threatening event^2^. Thus, developing a continual and accurate prediction model for in-hospital cardiac arrest in ICU settings is critical for enabling real-time detection and prompt interventions, including early defibrillation and cardiopulmonary resuscitation (CPR), to improve patient outcomes.

Numerous prediction models rely on electronic medical records (EMR) to extract various clinical features for predicting cardiac arrest. Generally, such models have good discrimination performance. A retrospective cohort study of patients with acute coronary syndrome collected 20 clinical variables such as vital signs, laboratory results, and electrocardiogram (ECG) reports within 24 h before cardiac arrest to develop prediction models, and the best model achieved a better discrimination performance than existing risk prediction scores, such as the National and Modified Early Warning scores^3^. Another study utilized EMR to collect nine clinical variables, including chief complaints and demographic data, to develop a prediction model for cardiac arrest in emergency departments^4^. Since such prediction models are often limited by the need to collect multiple variables from EMR, some of the variables may not be immediately available or reliable, while others may be completely unavailable in certain hospitals^5^. Contrarily, ECG, widely used for continuous monitoring of critically ill patients, accelerated by recent machine learning (ML) algorithms is capable of detecting various cardiac abnormalities automatically^6^. Therefore, an ECG-based prediction model can simplify the process and ensure constant, real-time monitoring for early and rapid prediction of in-hospital cardiac arrest in real-world clinical settings.

Several ECG-based markers such as heart rate, QRS prolongation, early repolarization, and heart rate variability (HRV) have been associated with in-hospital cardiac arrest, leading to the development of cardiac arrest prediction models that use these markers as predictors^7–9^. Among the aforementioned parameters, HRV, which is a measure of time variance between successive heartbeats (also known as RR intervals), has been identified as a promising predictor of cardiac arrest owing to its ability to evaluate the effects of autonomic nervous system activity in the heart^10–13^. Several HRV measures including the standard deviation of normal RR intervals (SDNN) and low-frequency (LF) and high-frequency (HF) powers have been reported as significant predictors of cardiac arrest^11,14^. Furthermore, a recent multi-center prospective cohort study suggested that HRV triangular index (HTI), calculated as the total number of RR intervals divided by their histogram height, can be an independent predictor of cardiac arrest^15^. However, these studies are limited by their focus on single HRV measures, overlooking the diverse information potentially offered by multiple HRV measures. Since all HRV measures originate from a single ECG source, the nature of HRV measures renders them cumbersome in conventional statistical models, including multivariable logistic regression.

The use of ML for developing prediction models has recently gained attention, as the models can learn complex relationships among several variables without requiring prespecified assumptions such as independence and linearity^16,17^. In a previous study^18^, a prediction model for heart failure was developed and validated using various 12-lead ECG features, including QT interval, QRS duration, R wave axis, T wave axis, and heart rate, along with demographic data, and the presence of atrial fibrillation (AF) and atrial flutter. This model achieved an impressive AUROC of 0.889. Lai et al. predicted sudden cardiac death by utilizing ECG-derived measurable arrhythmic risk, specifically three repolarization interval ratios and two conduction-repolarization markers^19^. However, the limited sample size (*n* = 46) restricts its broader implications, despite its exceptional accuracy of 99.49%. A recent review highlighted multiple ML-based prediction models for cardiac arrest using ECG^20^, but only two out of the 10 models used sample sizes exceeding 1000. One of these models, developed by Kwon et al., achieved an impressive AUROC of 0.948 for predicting cardiac arrest within 24 h, based on 12-lead ECG recordings from a substantial dataset of 25,672 patients^21^. The other model, created by Do et al. for predicting ventricular tachycardia, with an AUROC of 0.829, required a 3 h epoch of ECG data^22^. However, 12-lead ECG recordings may not be feasible for critically ill patients needing real-time monitoring. Therefore, HRV measures, easily obtainable from a single-lead ECG, have garnered attention. In a prospective observational study of 925 patients admitted to an emergency department, a support vector machine utilizing HRV measures with other clinical variables was found to be more accurate than a Modified Early Warning score in predicting cardiac arrest within 72 h, achieving an AUROC of 0.781^23^. Although these studies highlighted the potential of ML algorithms using HRV measures for predicting cardiac arrest, few studies have developed ML-based prediction models for in-hospital cardiac arrest using multiple HRV measures from only ECGs in large samples of critically ill patients.

In this study, we develop and validate an ML-based prediction model for in-hospital cardiac arrest in ICU patients using HRV. We collect ECG data from a large sample, single-center, retrospective cohort and extract various HRV measures. Thereafter, we utilize a modern ML algorithm to capture the complex relationship among these measures and improve the predictive performance. To the best of our knowledge, this study is the first to use ML models to predict in-hospital cardiac arrest in ICU patients, using multiple HRV measures as predictors, and to validate the model on a large sample of patients.

## Results

### Dataset construction

**Fig. 1:**
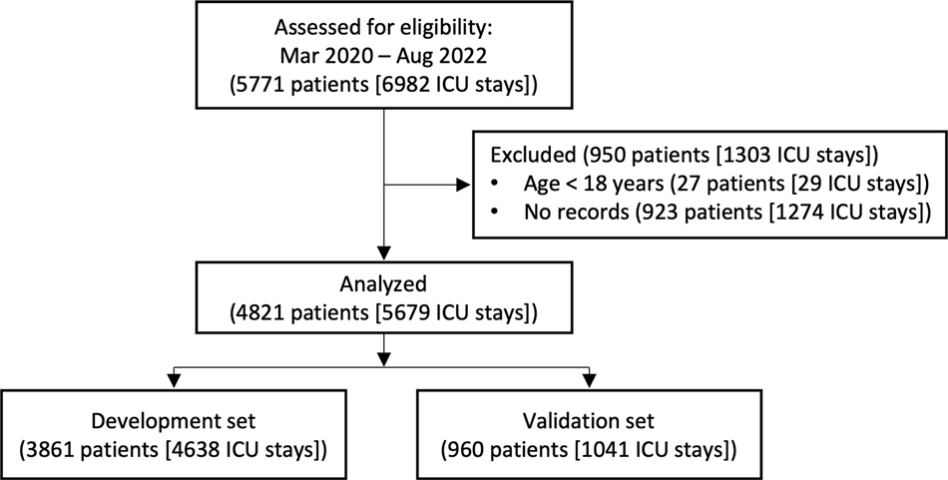
Flowchart of the study cohort. ICU intensive care unit.

A total of 5771 patients (6982 ICU stays) were eligible, of which 4821 patients (5679 ICU stays) were analyzed for developing and validating the proposed prediction model (Fig. 1). Patient demographics are listed in Table 1. The incidence of sudden cardiac arrest was 1.88%. The ECG data were preprocessed for quality checks, which resulted in 634,396 (1.24% event rate) and 139,663 (1.35% event rate) epochs in the development and validation sets, respectively. After the analysis of 43 HRV measurements, 33 were selected using the BorutaShap algorithm to develop the prediction model (Supplementary Fig. 1).

**Table 1:** Demographic characteristics of the study population at the ICU level.

	Study population (*n* = 5679)
Age (year)	62.6 ± 16.1
Sex (female, %)	2348 (41.3)
Height (cm)	163.1 ± 9.8
Weight (kg)	62.3 ± 13.3
Event of sudden cardiac arrest (*n*, %)	107 (1.88)
Type of intensive care unit	
Medical intensive care unit (*n*, %)	793 (14.0)
Surgical intensive care unit (*n*, %)	4886 (86.0)
Type of patient monitor	
SolarTM 8000 M (*n*, %)	828 (14.6)
IntelliVue (*n*, %)	4851 (85.4)

Data are presented as mean ± S.D. or number (%). *ICU* intensive care unit.

### Model evaluation results

**Fig. 2:**
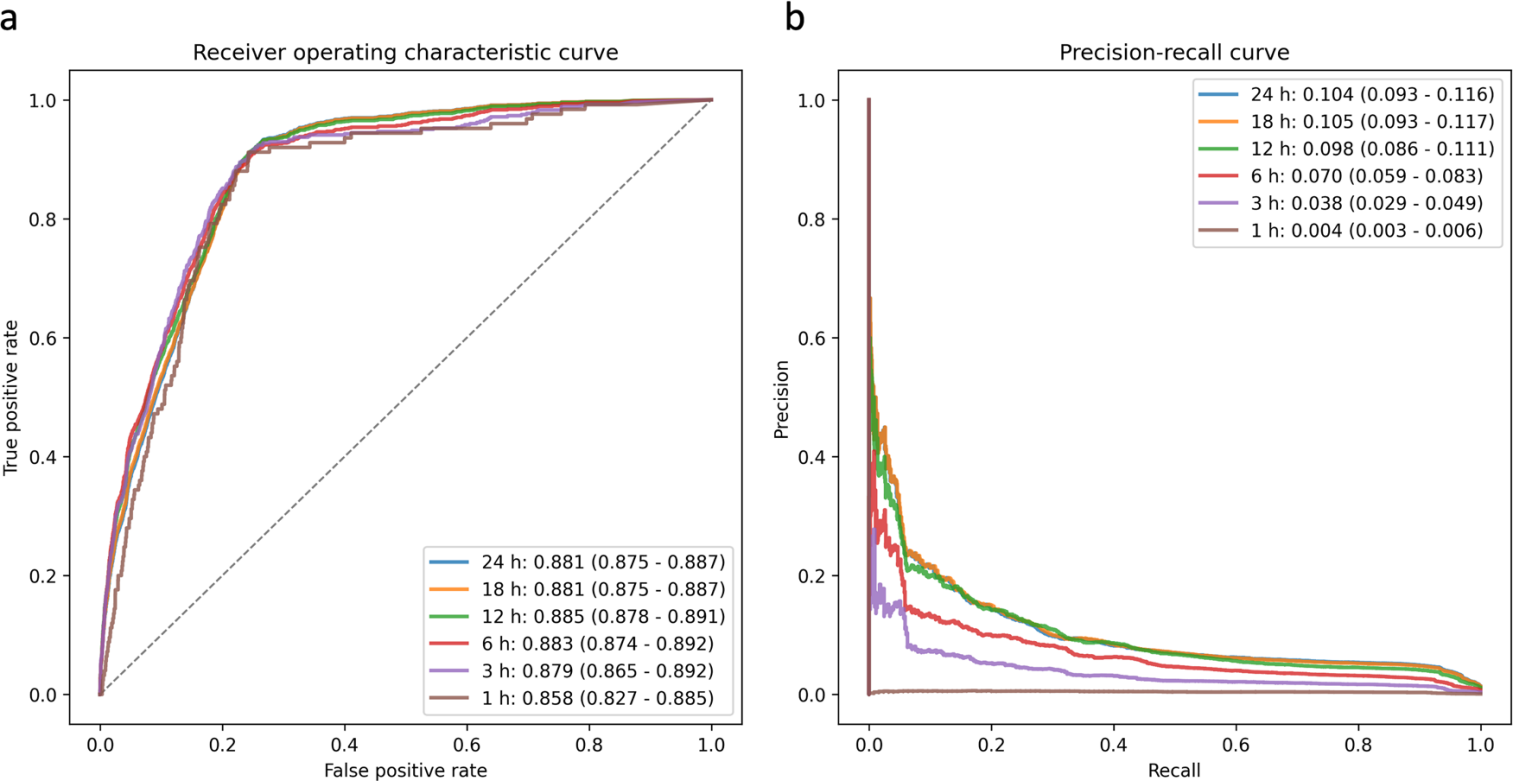
Receiver operating characteristic and precision-recall curves that represent the discrimination performance of the best model on the validation set. Each line shows the receiver operating characteristic curve (**a**) and the precision-recall curve (**b**) for predicting in-hospital cardiac arrest from 0.5 h to 24, 18, 12, 6, 3, and 1 h.

**Fig. 3:**
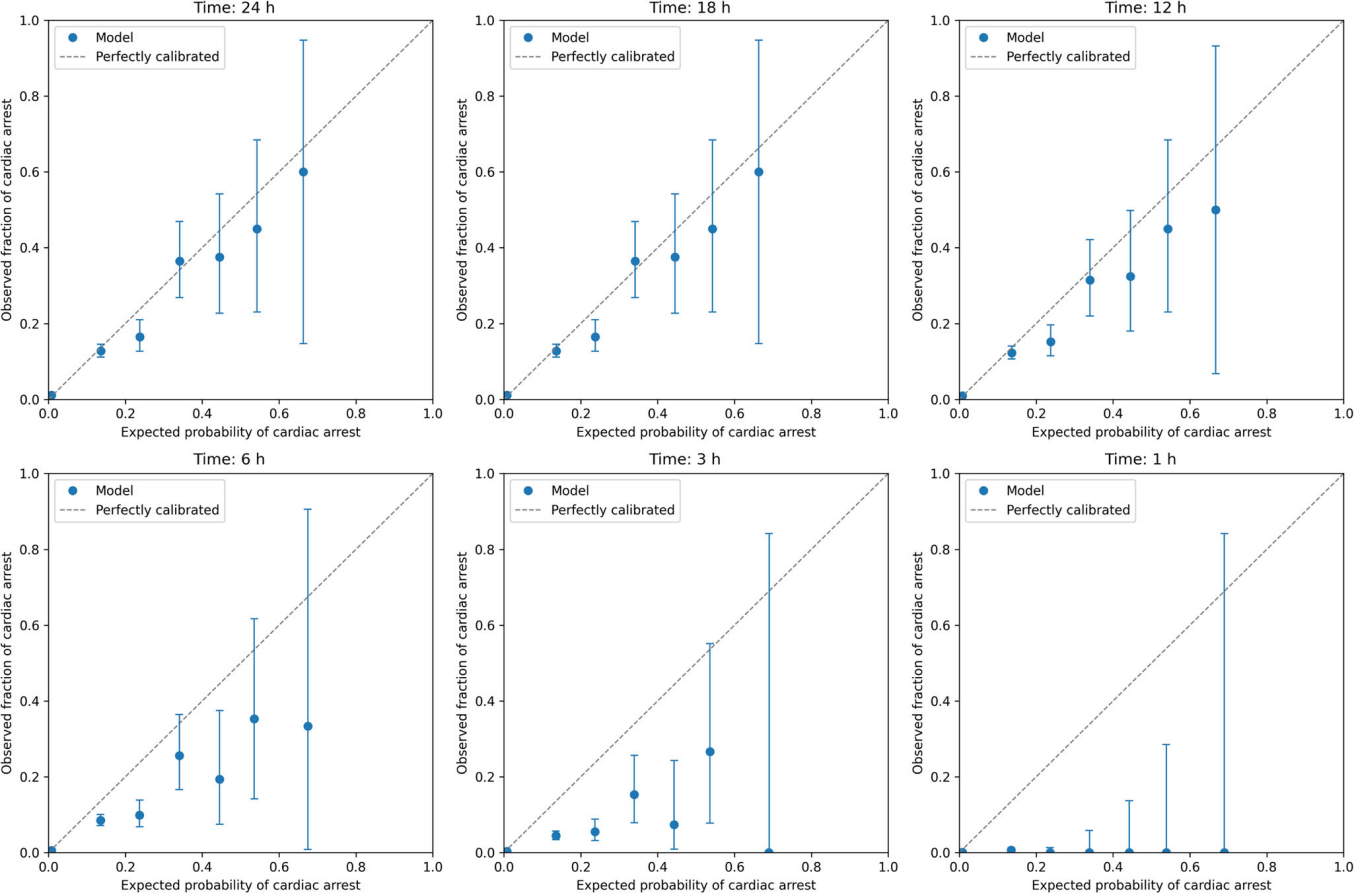
Calibration plot. The *x* and *y* axes represent the predicted probability and observed actual proportion of in-hospital cardiac arrest, respectively. The diagonal line represents a perfectly calibrated model, while deviations from this line indicate over- or under-prediction of in-hospital cardiac arrest. Error bars are 95% confidence intervals.

Following hyperparameter optimization through fivefold cross-validation, we retrained our light gradient boosting machine (LGBM) model on the entire development set and subsequently evaluated its performance on the validation set. As a primary outcome, the model achieved an area under the receiver operating curve (AUROC) of 0.881 [95% confidence interval (CI): 0.875–0.887] and an area under the precision-recall curve (AUPRC) of 0.104 [95% CI: 0.093–0.116] (Fig. 2). The AUROC of the secondary outcomes were comparable to that of the primary outcome, while the AUPRC declined as the range of the prediction period narrowed and neared the event of sudden cardiac arrest. Additional metrics assessing the discriminative performance of the model, including sensitivity, specificity, precision, accuracy, and F1-score, are presented in Table 2. Considering the calibration performance, our model overpredicted in the 0.2–0.3 range of predicted probability for the primary outcome (Fig. 3). For the secondary outcomes, our model exhibited consistent and reliable calibration at both 18 and 12 h. However, as the prediction period narrowed (from 6 to 1 h), the model increasingly overpredicted sudden cardiac arrests. The results of subgroup analyses, stratified by the type of patient monitor, are detailed in Table 3. The AUROCs of our model did not show significant variation between the two types of patient monitors.

**Table 2:** Discrimination performance of sudden cardiac arrest.

	AUROC	AUPRC	Sensitivity	Specificity	Precision	Accuracy	F1-score
Primary outcome							
Within 0.5–24 h	0.881 (0.875–0.887)	0.104 (0.093–0.116)	0.817 (0.800– 0.834)	0.800 (0.798–0.802)	0.053 (0.051–0.056)	0.809 (0.800–0.817)	0.100 (0.095–0.104)
Secondary outcomes							
Within 0.5–18 h	0.881 (0.875–0.887)	0.105 (0.093–0.117)	0.814 (0.796–0.832)	0.800 (0.798–0.802)	0.052 (0.049–0.054)	0.807 (0.798–0.816)	0.097 (0.093–0.102)
Within 0.5–12 h	0.885 (0.878–0.891)	0.098 (0.086–0.111)	0.827 (0.806–0.846)	0.800 (0.798–0.802)	0.044 (0.042–0.047)	0.813 (0.803–0.823)	0.084 (0.080–0.089)
Within 0.5–6 h	0.883 (0.874–0.892)	0.070 (0.059–0.083)	0.840 (0.817–0.862)	0.800 (0.798–0.802)	0.030 (0.028–0.032)	0.820 (0.809–0.831)	0.058 (0.055–0.062)
Within 0.5–3 h	0.879 (0.865–0.892)	0.038 (0.029–0.049)	0.850 (0.817–0.881)	0.800 (0.798–0.802)	0.016 (0.014–0.017)	0.825 (0.809–0.841)	0.031 (0.028–0.034)
Within 0.5–1 h	0.858 (0.827–0.885)	0.004 (0.003–0.006)	0.823 (0.755–0.888)	0.800 (0.798–0.802)	0.004 (0.003–0.004)	0.812 (0.778–0.844)	0.007 (0.006–0.009)

Data are presented as mean with 95% confidence interval. *AUROC* area under the receiver operating characteristic curve, *AUPRC* area under the precision-recall curve.

**Table 3:** Discrimination performance of sudden cardiac arrest stratified by patient monitor type.

	AUROC	AUPRC	Sensitivity	Specificity	Precision	Accuracy	F1-score
SolarTM 8000 M							
Within 0.5–24 h	0.867 (0.857–0.875)	0.039 (0.035–0.043)	0.749 (0.722– 0.777)	0.817 (0.815–0.819)	0.032 (0.030–0.034)	0.783 (0.769–0.797)	0.061 (0.057–0.066)
IntelliVue							
Within 0.5–24 h	0.853 (0.844–0.863)	0.259 (0.231–0.288)	0.887 (0.867–0.908)	0.692 (0.686–0.699)	0.125 (0.118–0.133)	0.790 (0.779–0.800)	0.220 (0.208–0.232)

Data are presented as mean with 95% confidence interval. *AUROC* area under the receiver operating characteristic curve, *AUPRC* area under the precision-recall curve.

### Comparative analysis

To provide context, a clinical parameter-based model was also developed for comparative purposes. All stages of developing the clinical parameter-based model, including feature selection, model development, and validation, mirrored those of our model. The BorutaShap algorithm selected 42 features, excluding only one (the difference feature of diastolic blood pressure). Our model showed a significantly higher AUROC than the clinical parameter-based model (0.881 vs. 0.735, *p* < 0.001). Additional metrics evaluating the discriminative performances of the model are presented in Supplementary Table 1.

### Feature importance analysis

**Fig. 4:**
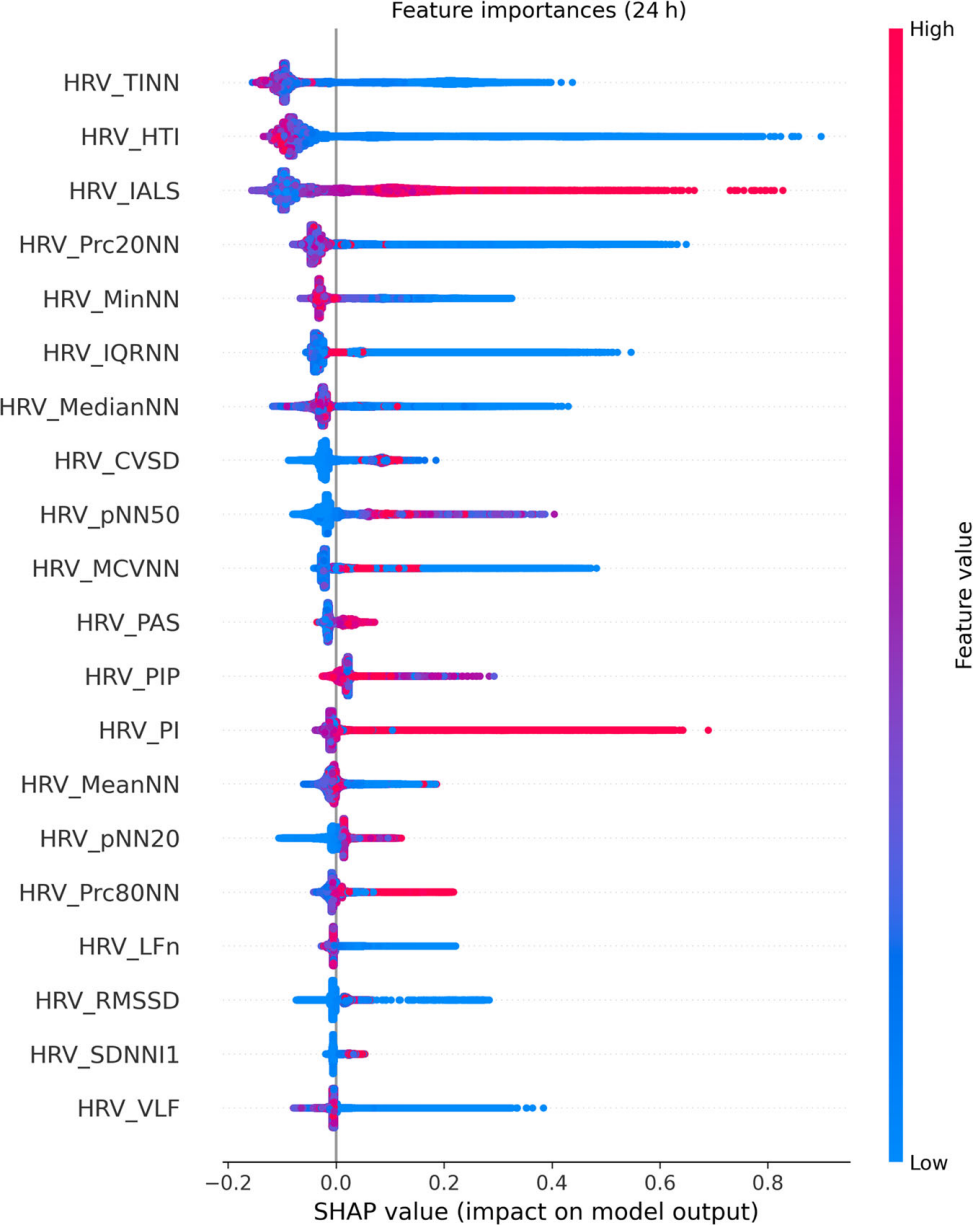
Shapley additive explanation dependence determines the relationship between the value of a feature and the predicted outcome of the model. Each dot represents a single prediction, while the *x* and *y* axes represent the mean absolute Shapley values and feature names, respectively. HRV heart rate variability, TINN baseline width of the triangular interpolation of the RR interval histogram, HTI heart rate variability triangular index, IALS inverse of the average length of the acceleration/deceleration segments, Prc20NN 20th percentile of the RR intervals, MinNN minimum of the RR intervals, IQRNN interquartile range of the RR intervals, MedianNN median of the RR intervals, CVSD root mean square of successive differences divided by the mean of the RR intervals, pNN50 proportion of RR intervals >50 ms, out of the total number of RR intervals, MCVNN median absolute deviation of the RR intervals divided by the median of the RR intervals, PAS percentage of NN intervals in alternation segments, PIP percentage of inflection points of the RR intervals series, PI Porta’s index, MeanNN mean of the RR intervals, pNN20 proportion of RR intervals >20 ms, out of the total number of RR intervals, Prc80NN 80th percentile of the RR intervals, LFn normalized low frequency, obtained by dividing the low-frequency power by the total power, RMSSD square root of the mean of the squared successive differences between adjacent RR intervals, SDNNI1 mean of the standard deviations of RR intervals extracted from 1-minute segments, VLF spectral power of very low frequencies, SHAP Shapley additive explanations.

The feature importance of our model was analyzed using the Shapley additive explanations (SHAP) method (Fig. 4). The most important feature, as determined by the SHAP values, was the baseline width of the triangular interpolation of the RR interval histogram (TINN), followed by HTI, the inverse of the average length of the acceleration/deceleration segments (IALS), 20th percentile of the RR intervals (Prc20NN), the minimum of the RR intervals (MinNN), and the interquartile range of the RR intervals (IQRNN). Among these features, a higher IALS value, as well as lower values of TINN, HTI, Prc20NN, and MinNN, were associated with a high risk of in-hospital cardiac arrest.

### Change of HRV measures over time until the event

**Fig. 5:**
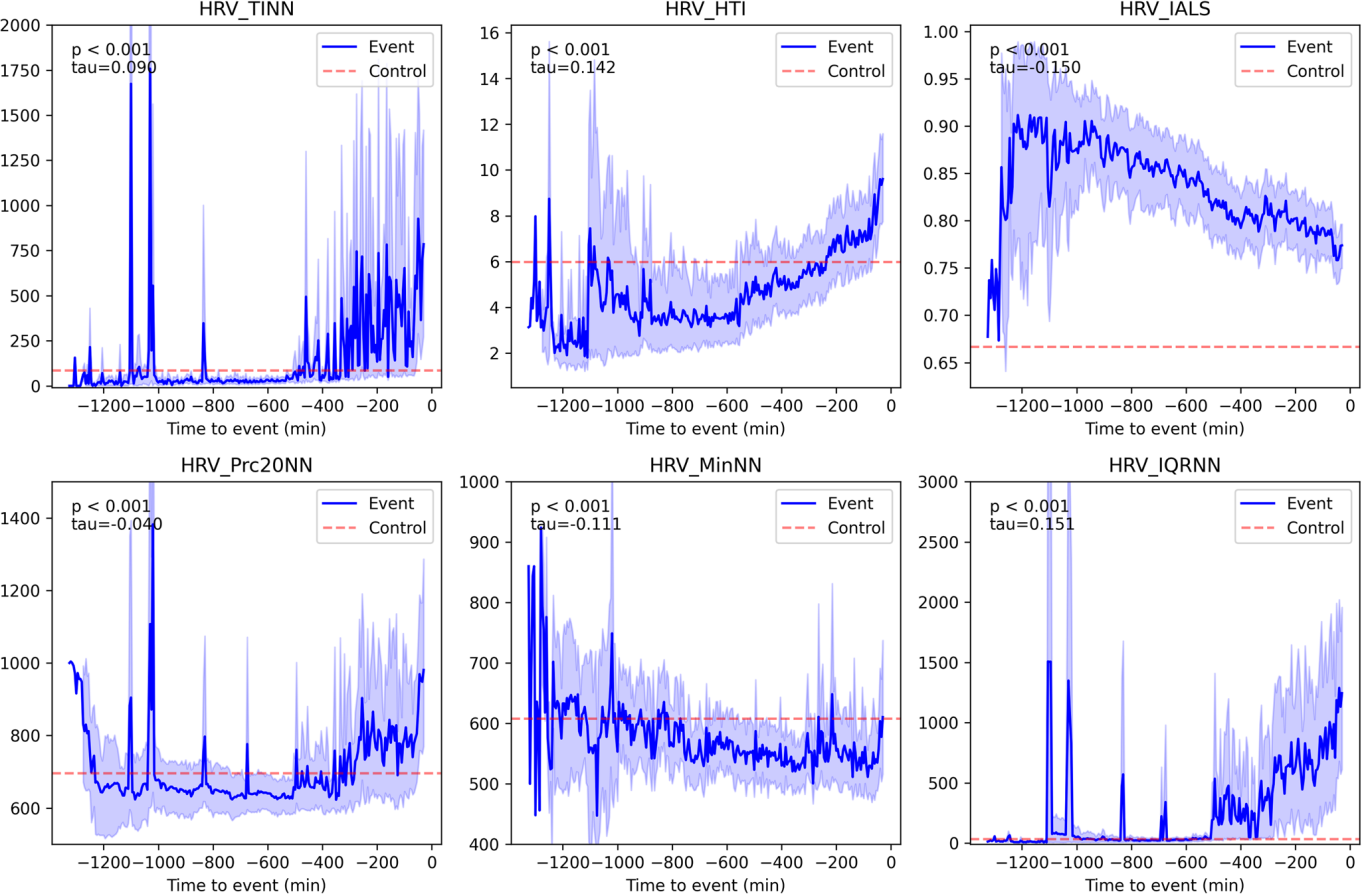
Changes in key HRV measures over time until the event. Fluctuations in the top six important HRV measures before in-hospital cardiac arrest are compared to their respective median values in patients without in-hospital cardiac arrest. The *x* and *y* axes represent time (min) and HRV measures values, respectively. The blue line and shaded region represent the mean value and 95% confidence intervals of HRV measures each time before the event of in-hospital cardiac arrest, respectively, while the red dashed line represents the median value of HRV measures in patients without in-hospital cardiac arrest. Kendall’s tau coefficient was used to measure the association between the time for the event and HRV measures. HRV heart rate variability, TINN baseline width of the triangular interpolation of the RR interval histogram, HTI heart rate variability triangular index, IALS inverse of the average length of the acceleration/deceleration segments, Prc20NN 20th percentile of the RR intervals, MinNN minimum of the RR intervals, IQRNN interquartile range of the RR intervals.

An additional analysis was conducted to reveal the changes in HRV measures before an in-hospital cardiac arrest, specifically within the timeframe of 0.5 h to 24 h preceding the cardiac arrest event. The results highlighted the top six important features in our model (Fig. 5). The HTI values increased until the event of cardiac arrest, whereas IALS and MinNN values decreased. TINN, Prc20NN, and IQRNN values started to increase at ~6 h before the event of cardiac arrest (Supplementary Fig. 2).

## Discussion

Recognizing the need for predicting in-hospital cardiac arrest in critically ill patients, we developed and validated an ML-based prediction model for in-hospital cardiac arrest using HRV measures in ICU patients. Our model leveraged HRV measures to overcome limitations encountered with conventional prediction models that rely on extensive EMR data. The proposed model not only simplifies the prediction process through a single data source but also facilitates real-time, continuous monitoring. The results demonstrated the potential of the LGBM model, which achieved good discrimination performance. This was paramount for the early detection and rapid prediction of in-hospital cardiac arrest, thereby improving patient outcomes in real-world clinical settings. This study highlights the (1) ability of the proposed model to predict the risk of in-hospital cardiac arrest using ECG data only, (2) usability of multiple HRV measures in the proposed ML-based model, and (3) explainability of the model through HRV measures.

In this study, only ECG data are used to predict the risk of in-hospital cardiac arrest, making our proposed model highly accessible and transferable to other healthcare settings that collect ECG data because continuous ECG monitoring is a standard practice in ICU settings. Unlike previous studies that employed multiple data sources, such as demographic information, vital signs, and laboratory results, to develop their prediction models^4,23–25^, our model finds easy application in clinical practice because only ECG data are required to predict cardiac arrest in ICU settings. Additionally, we conducted a comparative analysis between our model and a clinical parameter-based model from a previous study, which utilized 43 features derived from six vital signs. While the clinical parameter-based model achieved an impressive AUROC of 0.94 for predicting in-hospital cardiac arrest within 1 h, our findings indicated that its performance was not consistently maintained when predicting events occurring within 24 h (Supplementary Table 1).

Since HRV quantifies dynamic changes in ECG signals, previous studies utilized HRV measures to develop models in various medical contexts, including the prediction of poor outcomes or treatment responses^26–28^. However, such studies used traditional statistical models, such as a multivariable logistic regression model, which limited the number of HRV measures that could have been used owing to the linearity assumption between predictors and outcomes^29^. Contrarily, ML-based models handle complex relationships among predictors and outcomes, thus offering the advantage of using numerous other HRV measures, including IALS, pNN50, TINN, and HTI, in the model development process, in addition to the traditional sets of HRV measures, such as the mean of the RR intervals (meanNN), SDNN, LF, and HF^29^. Furthermore, ML-based models provide a distinct advantage while managing the inherently nonlinear and nonstationary fluctuations of HRV measures^29^. In our study, we utilized nonlinear measures including IALS, TINN, and HTI, which have been proven effective in detecting diseases such as end-stage renal disease, primary aldosteronism, and pulmonary hypertension^30–32^. The integration of these nonlinear HRV measures into ML algorithms proved to be of great potential in delivering superior discriminative performance. This observation was consistent with those of previous studies on different diseases^33^, thereby further endorsing the effectiveness of the proposed approach.

In this study, the BorutaShap algorithm was employed to identify the most relevant HRV measure from 43 HRV measures, resulting in the selection of 33 HRV measures as input features for the model. Utilizing such a comprehensive set of HRV measures increased the accuracy and robustness of the prediction model. The feature importance analysis results determined using the SHAP method revealed that TINN, HTI, IALS, Prc20NN, MinNN, and IQRNN were the most critical HRV measures in the in-hospital cardiac arrest prediction model.

TINN, standing as the most pivotal feature in our study, was closely followed by HTI. Both TINN and HTI are time-domain HRV measures derived from geometric analysis, providing insights into the overall shape and distribution of the RR interval histogram^10^. TINN quantifies the baseline width of the distribution of RR intervals using triangular interpolation, where the triangle is determined by the least squares error. A larger TINN value typically signifies greater variability in the RR intervals. Conversely, HTI reflects the total number of RR intervals divided by the height of these intervals, shedding light on how the RR intervals are distributed. A lower HTI suggests that a higher proportion of intervals cluster around the mode, while a higher HTI indicates a wider spread of intervals. Notably, previous research has emphasized the importance of both HTI and TINN in cardiac risk assessment. Studies have shown that these values tend to be significantly lower in patients with sudden cardiac death compared to those with hypertrophic cardiomyopathy or healthy individuals^34^. Additionally, in the context of developing prediction models for cardiac arrest in critically ill patients, TINN and HTI values have been found to be lower in patients experiencing cardiac arrest compared to those without^23^. These values have also exhibited distinctions in patients with arrhythmias compared to healthy individuals, with a notable difference in HTI values between these groups^35^. Furthermore, a previous study proved HTI to be an independent predictor of cardiovascular mortality in patients with AF^15^.

New HRV measures introduced in recent studies were applied to this study. A new HRV measure known as heart rate fragmentation or IALS was identified as one of the important features of our study. For IALS, acceleration, and deceleration segments were defined by a sequence of RR intervals between consecutive inflection points, for which the difference between the two RR intervals was <0 and >0, respectively. Segment length was determined as the number of RR intervals in that segment^36^. A prior study revealed that IALS was significantly higher in patients with congestive heart failure (CHF), with a mean IALS of 0.78. This result is similar to that of our study (Fig. 5), suggesting that higher IALS can be associated with compromised cardiac conditions^37^. Approximately 30–50% of the patients with CHF were estimated to be at risk of sudden cardiac arrest^38^.

Few studies have used the other HRV measures included in our study, such as IQRNN, to study the relationship between those measures and cardiac arrest; however, our findings suggested that IQRNN has the potential as predictors of cardiac arrest. The values of IQRN, as well as TINN in patients experiencing sudden cardiac arrest, remained similar to those in patients without sudden cardiac arrest up until approximately 6 h prior to the event, after which dynamic changes occurred. Nevertheless, the causality between these HRV measures and cardiac arrest requires further investigation.

Changes in HRV measures were analyzed within the timeframe of 0.5 h to 24 h preceding the in-hospital cardiac arrest and compared with their median values in patients without in-hospital cardiac arrest, as shown in Fig. 5. The IALS values were consistently higher within 0.5 to 24 h preceding the cardiac arrest event compared to the patients without in-hospital cardiac arrest; however, there was a decreasing trend in these values leading up to the cardiac arrest event. Conversely, HTI values started low, but increased towards the event of cardiac arrest. These consecutive changes in HRV measures have not been documented in previous studies. Therefore, the analytical results of this study are expected to provide valuable insights into the real-time condition evaluation of a patient and facilitate the prompt initiation of interventions aimed at preventing events of cardiac arrest.

Furthermore, this study has the significant advantage of utilizing a large sample size of ~5000 patients, which adds to the representativeness and generalizability of the results to other patient populations. A large sample size is critical for accurately detecting rare events, such as in-hospital cardiac arrest, which is essential for developing reliable ML-based predictive models^39,40^.

Nevertheless, the limitations of this study must be considered while interpreting the results. The binary classification model used has certain restrictions; the model can only predict whether a patient will experience a cardiac arrest but does not provide information on the timing of the event; however, we tried to evaluate our model on different time periods as secondary outcomes. Additionally, the model does not account for the influence of treatment interventions on outcomes and focuses solely on baseline predictors. The selection of the development and validation sets may also have been biased, which can affect the accuracy and generalizability of the results. Furthermore, the study was performed at a single center, limiting the transferability of the findings to other patient populations and healthcare systems.

Future research should focus on validating the findings of this study in larger multi-center studies to increase the generalizability of the results and confidence in the predictions made by the model. Open datasets with labels for cardiac arrest and ECG waveforms, such as the Medical Information Market for Intensive Care, can help validate our results before conducting a multi-center prospective study. Moreover, incorporating clinical factors such as comorbidities or medications may further assist the model^41^; however, we intentionally excluded these factors in this study considering variable availability across different hospital settings. Additionally, developing survival models that account for both the probability and timing of a cardiac arrest event is expected to provide valuable information for clinical decision-making and allow a better understanding of the long-term outcomes of patients who experience sudden cardiac arrest in ICU settings.

In conclusion, we developed and validated an ML-based real-time prediction model to predict in-hospital cardiac arrest in critically ill patients, focusing on the importance of HRV measures. If future prospective studies validate our results, they can potentially be used to detect in-hospital cardiac arrest in critically ill patients.

## Methods

### Study design

All data for model development were retrieved from a prospective registry containing vital signs of ICU patients at the Seoul National University Hospital (SNUH). The prospective registry was approved by the Institutional Review Board (IRB) of SNUH (approval number: 1408-101-605) and registered at ClinicalTrials.gov (NCT02914444). Furthermore, the IRB approved the retrospective analysis of data from the prospective registry (approval number: 2303-113-1413). Due to the retrospective nature of this study and the anonymity of data, the IRB waived off the requirement for written informed consent from patients.

### Data collection

For this study, registry data of all the patients admitted to medical or surgical ICUs (MICU or SICU) at SNUH from March 2020 to August 2022 were eligible. However, patients under the age of 18 and those without ECG recordings were excluded from the study. The ECG data used in this study were collected using two different patient monitors (IntelliVue, Philips Healthcare, Amsterdam, Netherlands, and SolarTM 8000 M, GE Healthcare, Wauwatosa, WI, USA) and stored in a free biosignal collection program (version 1.9.9, accessed on June 6, 2022, https://vitaldb.net)^17^. The clinical parameters, used for comparative analysis, including heart rate, systolic blood pressure, diastolic blood pressure, mean blood pressure, SpO_2_, and respiration rate, were also collected using the same patient monitors. The event time of CPR was extracted from the clinical data warehouse of SNUH (Supreme 1.0, Seoul National University Hospital, Seoul, Republic of Korea) to incorporate the presence and time of sudden cardiac arrests during each ICU stay. For ICU stays with multiple CPR attempts, only the first CPR event was used.

**Fig. 6:**
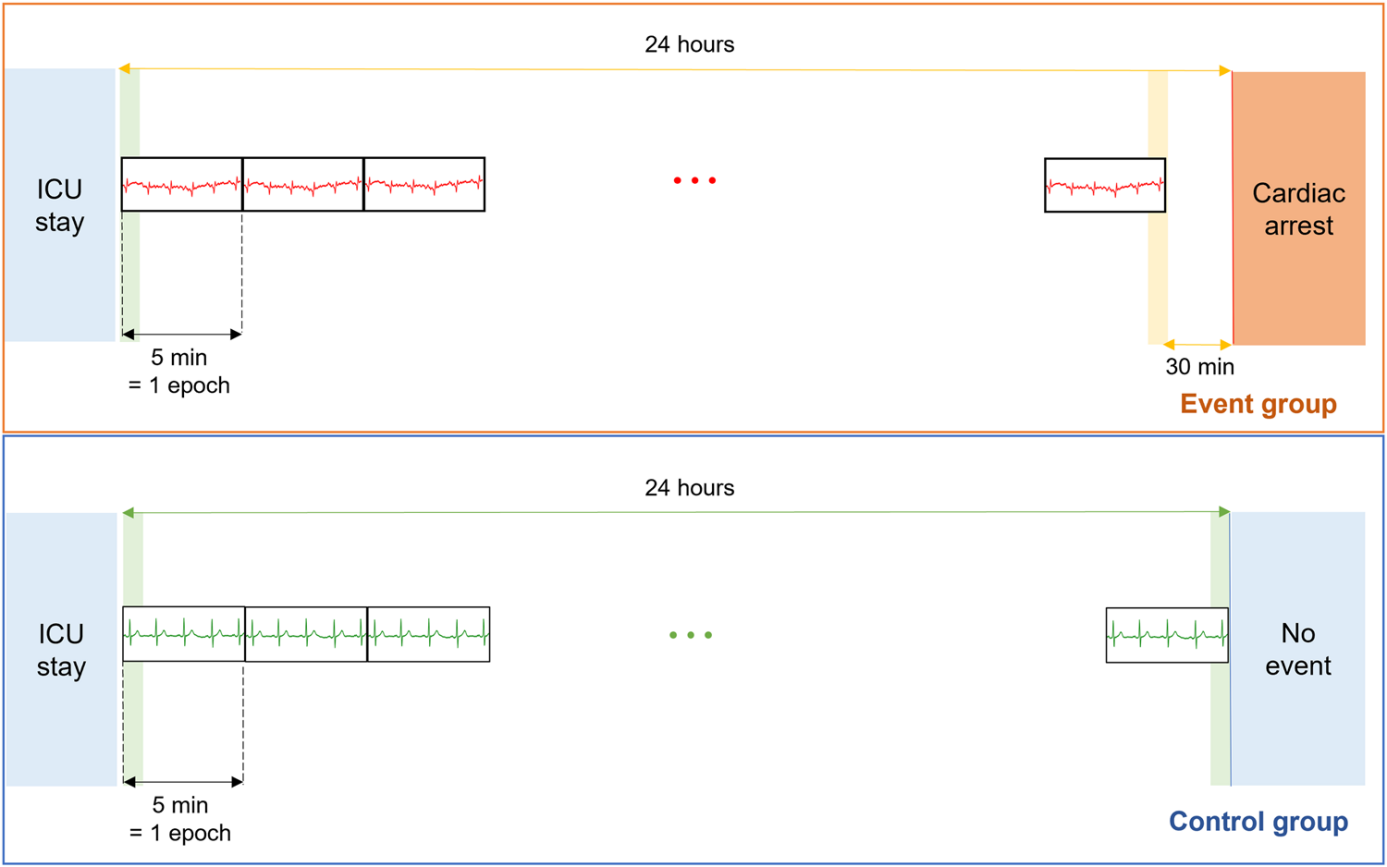
Collection protocol of 5 min epoch within ECG data. ICU intensive care unit.

We constructed a structured ECG dataset of ICU stays with (event group) and without (control group) sudden cardiac arrests (Fig. 6). For the event group, ECG data from 0.5 to 24 h prior to the event were collected and 5 min epochs with 5 min intervals spanning 0.5–24 h were extracted. Each epoch begins immediately after the end of the previous one. For the control group, we randomly sampled ECG data for 24 h from each ICU stay and extracted 5 min epochs with 5 min intervals similar to the event group. Only data from the 5-min epochs were used in calculating the HRV features and predicting the event. Thereafter, the dataset was randomly divided into development (80%) and validation (20%) sets at the patient level, while maintaining the same ratio of groups in both sets.

### Data preprocessing

The ECG signals were originally collected at a sampling rate of 500 Hz but were downsampled to 250 Hz to reduce the amount of data and computational resources required for processing. To ensure a more accurate HRV analysis, a series of preprocessing steps were applied to the ECG data: ECG signals were divided into 5 min epochs, signals were filtered to remove noise, and data quality was checked to ensure that the data were usable. Specifically, a 0.5 Hz high-pass Butterworth filter (order = 5) was used, followed by powerline filtering of the 5 min ECG signal during the first step. Next, a continuous quality index was computed by interpolating the distance of each QRS segment from the average QRS segment (1 corresponded to the heartbeats closest to the average sample, while 0 corresponded to the most distant heartbeat from the average sample). When the proportion of QRS segments with a quality index greater than or equal to 0.9 was not greater than 80%, a few 5 min epochs were excluded according to the quality index. For the clinical parameter-based model, which was developed based on a prior study^25^, we initially established a total of 43 features. This set included six raw features, six differential features, 30 statistical features computed using a sliding window approach, and one additional feature known as the shock index. Because the clinical parameters were originally collected at 2 sec intervals, we extracted median values within each 5 min epoch for the raw features. The differential features were calculated as the differences between the values in the current epoch and those in the previous epoch for the clinical parameters. We applied a fixed-length 2-h sliding window to segment each parameter with a 5 min interval. These segments were then aggregated to statistical measures such as the mean, median, minimum, maximum, and standard deviation for each feature across all the segments.

### Calculation of HRV parameters

The Neurokit2 Python library, a comprehensive and validated toolkit for ECG signal analysis, was utilized to detect R peaks and extract various HRV measures from each 5 min epoch, as employed in previous studies^42–44^. The toolkit facilitated the calculation of HRV measures based on detected R peaks, ensuring reliable results through a standardized and automated approach. The HRV measures were calculated using the detected R-peak information. The toolkit provided a total of 74 HRV measures comprising 24 time-domain measures such as meanNN, SDNN, and square root of the mean of the squared successive differences between adjacent RR intervals (RMSSD); 9 frequency-domain measures such as the spectral power of LF, HF, and the ratio of LF to HF (LF/HF); and 41 nonlinear measures such as the standard deviation perpendicular to the line of identity (SD1), cardiac sympathetic index, and cardiac vagal index. In the nonlinear category, we preselected 15 measures, which were derived either from the Poincaré plot^45,46^, or the heart rate fragmentation approach^36^. Additionally, we excluded four time-domain HRV measures as they required ECG epochs longer than 5 min. Consequently, we began with a total of 43 HRV features as the initial feature candidates before applying the BorutaShap algorithm (Supplementary Fig. 1).

### Measurement outcome

The primary outcome was the occurrence of cardiac arrest within 0.5–24 h, as in a previous study^3,4^. The secondary outcomes included the occurrences of cardiac arrest from 0.5 to 18, 12, 6, 3, and 1 h. The discrimination performances of the model were evaluated using AUROC, AUPRC, sensitivity, specificity, precision, accuracy, and F1-score. To evaluate the calibration performance of the model, we graphed a calibration plot comparing the predicted probabilities of sudden cardiac arrest against the observed fractions.

### Feature selection

For feature selection, we employed the BorutaShap algorithm because it uses a combination of the Boruta and SHAP algorithms to identify the most important features in a given dataset^47^. Thus, the BorutaShap algorithm was applied to identify the most relevant features from the 43 predetermined HRV measures extracted for our model, and 43 clinical features extracted for the clinical parameter-based model, respectively. In this study, only the features categorized as “accepted” by the BorutaShap algorithm were chosen for inclusion in the model development process, with those marked as “tentative” or “rejected” being excluded.

### Model development and validation

A LGBM model was utilized to develop the proposed ML-based prediction model, which is an implementation of the decision tree-based ensemble algorithm, with high efficiency, scalability, and strong performance on a wide range of datasets^48^. The hyperparameters of the LGBM model were optimized using Bayesian optimization, which is an efficient approach to automatically tune ML algorithms by modeling the generalization performance of a learning algorithm as a sample from a Gaussian process and using the tractable posterior distribution to select the next optimal parameter for trial^49^. Hyperparameter optimization was conducted with fivefold cross-validation at the patient level using the development set to find the best hyperparameters such as the number of leaves, fraction of features, and regularization determined using the AURPC score. Subsequently, we performed another round of fivefold cross-validation at the patient level to identify the optimal model training parameters including the number of boosting iterations. Once these optimal parameters were established, we proceeded to train the model using the entire development set. Following this, we implemented the Beta calibration method for further refinement^50^. Following this, we implemented the Beta calibration method for further refinement. The final model, resulting from these steps, was tested with the validation set. All this learning scheme was applied to both our model and the clinical parameter-based model.

### Feature importance

We employed the SHAP method^51^ to explain the output of the ML model based on a game-theoretic framework. Each feature was assigned a unique contribution value that indicated its impact on the prediction outcome. In the classic concept of Shapley values from cooperative game theory, SHAP values are grounded and a way to distribute the prediction outcomes fairly among all features is provided. Additionally, this method is used to determine the attributes of each feature to the predicted outcome applied to the validation set.

### Statistical analysis

Kendall’s tau coefficient was used to measure the association between the time for the event and HRV measures. DeLong’s test was employed to compare the AUROCs^52^. All statistics were reported with point estimates and 95% CIs. Python 3.8.0 (Python Software Foundation, Wilmington, DE, USA) was used for signal preprocessing, model development, validation, statistical testing, and visualization. A *p* value < 0.05 was considered statistically significant.

### Supplementary information

Supplementary Material

## Data Availability

The dataset used in this study is not publicly available. However, the data of this study can be provided if there is a reasonable request to the corresponding author.

## Code availability

The code to generate the result of this study can be accessed at https://github.com/HyeonhoonLee/hrvarrest.
