# Supplementary material for: Real-time machine learning model to predict in-hospital cardiac arrest using heart rate variability in ICU
Source: NPJ Digit Med. 2023 Nov 23;6:215. doi: 10.1038/s41746-023-00960-2 (PMC10665411; doi:10.1038/s41746-023-00960-2)

Supplementary Table 1. Discrimination performance of cardiac arrest in a clinical parameter-based model.

	AUROC	AUPRC	Sensitivity	Specificity	Precision	Accuracy	F1-score
Primary outcome							
Within 0.5-24h	0.735 (0.720-0.749)	0.058 (0.051-0.065)	0.460 (0.434-0.484)	0.881 (0.879-0.883)	0.040 (0.037-0.043)	0.671 (0.658-0.683)	0.073 (0.068-0.079)
Secondary outcomes							
Within 0.5-18h	0.744 (0.729-0.757)	0.059 (0.051-0.066)	0.473 (0.447-0.499)	0.881 (0.879-0.883)	0.040 (0.037-0.043)	0.677 (0.664-0.690)	0.073 (0.068-0.079)
Within 0.5-12h	0.760 (0.743-0.777)	0.055 (0.047-0.062	0.525 (0.495-0.554)	0.881 (0.879-0.883)	0.036 (0.033-0.039)	0.703 (0.688-0.718)	0.067 (0.062-0.073)
Within 0.5-6h	0.772 (0.753-0.792)	0.045 (0.038-0.053)	0.585 (0.550-0.621)	0.881 (0.879-0.883)	0.027 (0.025-0.030)	0.733 (0.715-0.751)	0.052 (0.048-0.057)
Within 0.5-3h	0.776 (0.749-0.804)	0.026 (0.020-0.033)	0.611 (0.560-0.659)	0.881 (0.879-0.883)	0.015 (0.014-0.017)	0.746 (0.721-0.770)	0.030 (0.026-0.034)
Within 0.5-1h	0.841 (0.795-0.881)	0.008 (0.005-0.012)	0.613 (0.515-0.711)	0.881 (0.879-0.883)	0.004 (0.003-0.005)	0.747 (0.698-0.796)	0.008 (0.006-0.010)

Data are presented as mean with 95% confidence interval. AUROC: area under the receiver operating characteristic curve; AUPRC: area under the precision-recall curve.

Supplementary Figure 1. Heart rate variability parameters accepted and rejected by the BorutaShap method. HRV: heart rate variability; IALS: inverse of the average length of the acceleration/deceleration segments; pNN50: proportion of RR intervals greater than 50ms, out of the total number of RR intervals; TINN: baseline width of the triangular interpolation of the RR interval histogram; HTI: heart rate variability triangular index; PI: Porta's index; Prc20NN: 20th percentile of the RR intervals; pNN20: proportion of RR intervals greater than 20ms, out of the total number of RR intervals; MCVNN: median absolute deviation of the RR intervals divided by the median of the RR intervals; SDNNI1: mean of the standard deviations of RR intervals extracted from 1-minute segments; MinNN: minimum of the RR intervals; CVSD: root mean square of successive differences divided by the mean of the RR intervals; VHF: spectral power of high frequencies; RMSSD: square root of the mean of the squared successive differences between adjacent RR intervals; PSS: percentage of short segments; LFn: normalized low frequency, obtained by dividing the low frequency power by the total power; CSI: Cardiac sympathetic index; MeanNN: mean of the RR intervals; PIP: percentage of inflection points of the RR intervals series; IQRNN: interquartile range of the RR intervals; HFn: normalized high frequency, obtained by dividing the high frequency power by the total power; SD1: Standard deviation perpendicular to the line of identity; SD2: Standard deviation along the identity line; SD1SD2: ratio of SD1 to SD2; LF: spectral power of low frequencies; SI: slope index; MaxNN: maximum of the RR intervals; VLF: spectral power of very low frequencies; SDANN1: standard deviation of average RR intervals extracted from 1-minute segments; Prc80NN: 80th percentile of the RR intervals; PAS: percentage of NN intervals in alternation segments; CSI_Modified: modified cardiac sympathetic index, obtained by dividing the square of the longitudinal variability by its transverse variability; LFHF: ratio obtained by dividing the low frequency power by the high frequency power; AI: Area index; S: area of ellipse described by SD1 and SD2; GI: Guzik's index; SDNN: standard devication of the RR intervals; HF: spectral power of high frequencies; SDSD: standard deviation of the successive differences between RR intervals; MadNN: median absolute deviation of the RR intervals; LnHF: the log transformed spectral power of high frequencies; CVI: cardiac vagal index.

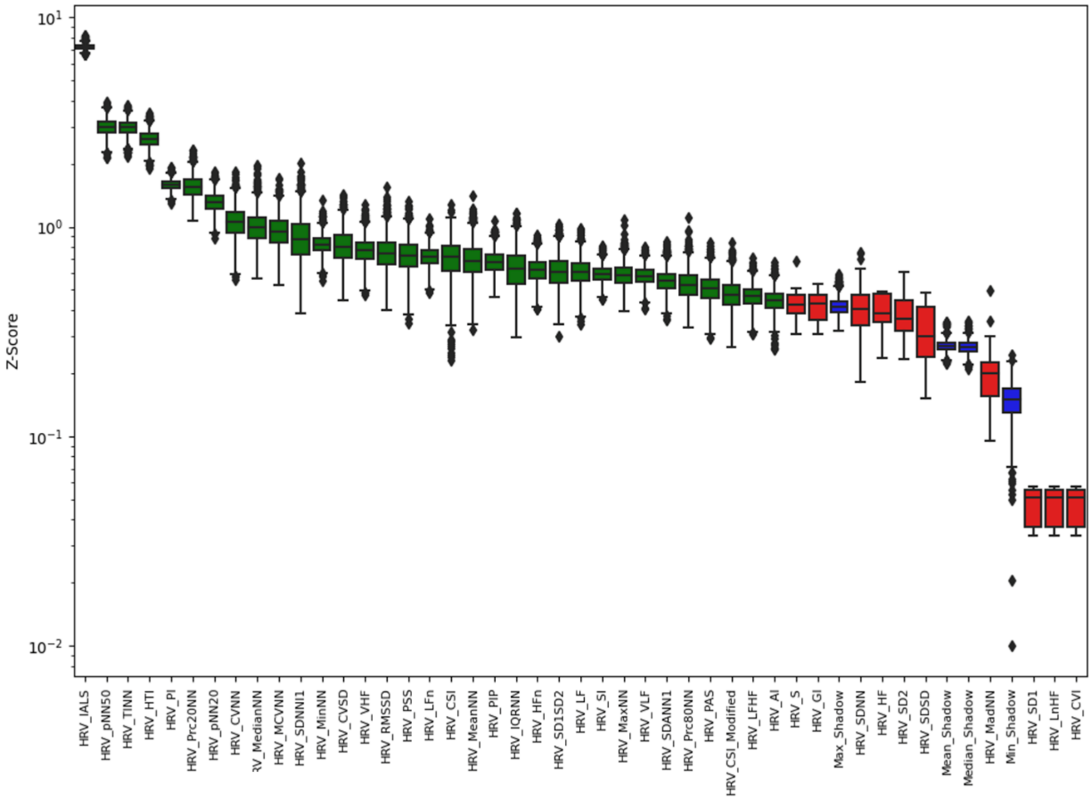

Supplementary Figure 2. Changes in key heart rate variability (HRV) measures over time until the event (6 – 0.5 h). Fluctuations in the top six important HRV measures before in-hospital cardiac arrest (6 – 0.5 h) are compared to their respective median values in patients without in-hospital cardiac arrest. The x and y axes represent time (min) and HRV measures values, respectively. The blue line and shaded region represent the mean value and 95% confidence intervals of HRV measures of each time before the event of in-hospital cardiac arrest, respectively, while the red dashed line represents the median value of HRV measures in patients without in-hospital cardiac arrest. Kendall's tau coefficient was used to measure the association between the time for the event (6 – 0.5 h) and HRV measures. HRV: heart rate variability; TINN: baseline width of the triangular interpolation of the RR interval histogram; HTI: heart rate variability triangular index; IALS: inverse of the average length of the acceleration/deceleration segments; Prc20NN: 20th percentile of the RR intervals; MinNN: minimum of the RR intervals; IQRNN: interquartile range of the RR intervals.

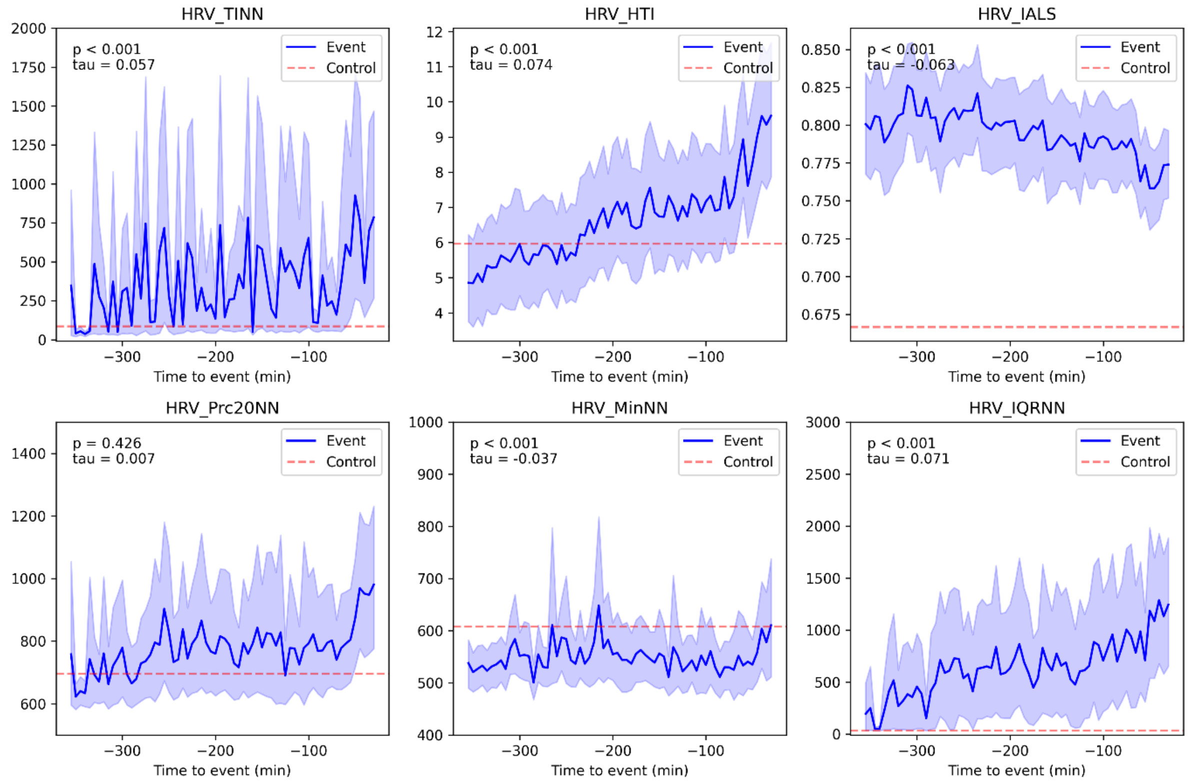

Supplement: Supplementary file 1 — Supplementary Material [file 41746_2023_960_MOESM1_ESM.pdf]
